# GPU-acceleration of the distributed-memory database peptide search of mass spectrometry data

**DOI:** 10.1038/s41598-023-43033-w

**Published:** 2023-10-31

**Authors:** Muhammad Haseeb, Fahad Saeed

**Affiliations:** 1 Knight Foundation School of Computing and Information Sciences, Florida International University (FIU), Miami, FL USA; 2 Biomolecular Sciences Institute (BSI), Miami, FL USA; 3 Department of Human and Molecular Genetics, Herbert Wertheim School of Medicine, Florida International University, Miami, FL USA

**Keywords:** Proteomics, Computational biology and bioinformatics

## Abstract

Database peptide search is the primary computational technique for identifying peptides from the mass spectrometry (MS) data. Graphical Processing Units (GPU) computing is now ubiquitous in the current-generation of high-performance computing (HPC) systems, yet its application in the database peptide search domain remains limited. Part of the reason is the use of sub-optimal algorithms in the existing GPU-accelerated methods resulting in significantly inefficient hardware utilization. In this paper, we design and implement a new-age CPU-GPU HPC framework, called *GiCOPS*, for efficient and complete GPU-acceleration of the modern database peptide search algorithms on supercomputers. Our experimentation shows that the GiCOPS exhibits between 1.2 to 5$\times$ speed improvement over its CPU-only predecessor, HiCOPS, and over 10$\times$ improvement over several existing GPU-based database search algorithms for sufficiently large experiment sizes. We further assess and optimize the performance of our framework using the Roofline Model and report near-optimal results for several metrics including computations per second, occupancy rate, memory workload, branch efficiency and shared memory performance. Finally, the CPU-GPU methods and optimizations proposed in our work for complex integer- and memory-bounded algorithmic pipelines can also be extended to accelerate the existing and future peptide identification algorithms. GiCOPS is now integrated with our umbrella HPC framework HiCOPS and is available at: https://github.com/pcdslab/gicops.

## Introduction

Identification of peptides from the mass-spectrometry (MS) data is a critical step in computational proteomics^1–4^. Database peptide search is the most commonly employed computational technique to deduce peptide sequences from the large volumes of experimentally generated MS spectra data^5–8^. In database search, the experimental spectra are searched against a database of indexed theoretically simulated spectra, to identify the most similar and confident match(es)^3^. The similarity between a pair of experimental and theoretical spectrum is computed using one or a combination of distance metrics such as cross-correlation (xcorr) score^1,9^, hyperscore^4,7^, and Z-score^10^. More recently, *open-search*-based scoring techniques have been used to identify known and unknown *post-translational modifications* (PTMs) at the expense of exponential increase space and time requirements^4^. We have introduced both algorithmic^11^, and high-performance computing solutions^4^ to drastically reduce the run times for these experiments from days into hours and minutes.

Graphics Processing Units (GPUs) have emerged as the primary and ubiquitous hardware accelerator in the current-generation of (top-500) supercomputers^12,13^. As the high-performance computing (HPC) paradigm continues to shift towards heterogeneous computing, GPU based algorithms are increasingly developed to speed up the complex algorithms across several scientific domains^14–17^. However, the existing GPU-based database search algorithms and software including Tempest^18^, Tide-for-PTM-search^19^, GPUScorer^20^, ProteinByGPU^21^, GPU based SDP^22^, and MIC-Tandem^23^ are designed to accelerate only the *spectral scoring* computations and do not leverage or accelerate the advanced reductive (pre-screening) algorithms such as fragment-ion indexing and matching^7,24^ and sequence-tagging^6,25^. This results in excessive on-the-fly trivial computations, resulting in the existing methods to exhibit sub-optimal performance, especially for *open-search* where the precursor-mass filters are wide^26^ (Supplementary Sections 1 and 2).

The need for efficient GPU-accelerated database peptide search algorithms stems from the computational demands of large-scale proteomics pipelines^4^, the post-Moore technological shift towards heterogeneous computing^12^, and the increasing imbalance between the MS-data generation and database search speeds.

In this paper, we design and implement GPU-accelerated algorithms and software for scalable acceleration of the database peptide search on CPU-GPU architectures. We integrate our developed techniques with the HiCOPS’s high-performance computing (HPC) software framework to also accelerate the database search across the distributed-memory supercomputing nodes. Our new framework, called GiCOPS (GPU-accelerated HiCOPS), provides orders-of-magnitude speed improvement over its CPU-only predecessor i.e., HiCOPS, as well as several existing GPU-based database peptide search algorithms for *open-search* application. The proposed GiCOPS algorithms leverage reduction trees, lock-free computational strategies, shared memory computations, and iterative performance tuning optimizations to scalably accelerate the complex integer- and memory-intensive computations of the fragment-ion index-based^4,7,24^ database peptide search on GPU.

Our extensive experiments show that GiCOPS outperforms the existing GPU-based database search algorithms by more than 10$\times$ in both closed- and open-search modes. Our experimentation also shows that the GiCOPS provides a speed improvement of 1.2 to 5 $\times$ as compared to its CPU-only predecessor (HiCOPS) while providing identical peptide identifications. Finally, our comprehensive performance analysis and optimization using NVIDIA Nsight Compute (NCU) profiler and the Instruction Roofline Model^27^ show achieved performance of 143.6 warp giga instructions per second (GIPS) (48% theoretical peak) and 64.65 warp GIPS (21.5% theoretical peak) for open- and closed- database search respectively on NVIDIA RTX A6000 GPU.

## Results

### Methods overview

GiCOPS accelerates the core computational algorithms in the database peptide search workflow, and implements optimizations and CPU-GPU pipelines to efficiently utilize all available compute and memory resources. The database peptide search workflow consists of four main steps: (1) database construction, (2) experimental MS data pre-processing, (3) database peptide search and (4) post-processing. In the first step (database construction), the peptide sequence database is offloaded to the GPU where the theoretical spectra are generated, indexed and communicated back to the CPU. In the second step (data pre-processing), the experimental MS data are streamed to the GPU where it is cleaned, pre-processed, and communicated back to the CPU. In the third step (database peptide search), the entire database index is communicated to the GPU’s RAM and the experimental MS data are streamed in batches. GPU searches each batch against the in-memory database index. In the fourth step (post-processing), the results are used to compute expected values and confidence scores, which are finally communicated to the CPU. At each step, a priority-queue based work stealing pipeline is implemented to simultaneously offload the *ready* compute work at both the CPU and GPU depending on their priority and availability (Supplementary Fig. 1). The underlying algorithms are redesigned to efficiently exploit the GPU architectures and are expressed in terms of building block kernels, each optimized using multi-level reductions, lock-free synchronizations, register-exchange based thread communications, and shared memory computations for maximum throughput. Finally, to accelerate across distributed-memory supercomputers, GiCOPS employs the HiCOPS’s^4^ *four* Bulk Synchronous Parallel (BSP) superstep^28^ design where all parallel nodes asynchronously execute each superstep while synchronizing between supersteps. Since all nodes execute the same code, the rest of the paper will discuss the GiCOPS methods and results for a single-node CPU-GPU machine. GiCOPS requires the minimum number of GPUs ($G_{min}$) to be $\ge D/M_{g}$; where *D* is the database size and $M_{g}$ is the GPU DRAM.

The total wall time ($T_{G}$) for executing the four GiCOPS steps is the sum of individual steps (*j*) and each step’s time ($T_{j}$) is the maximum time required by the (simultaneously operating) CPU-side to complete its computational work ($T_{j,c}$) or the GPU-side to complete its scheduled computational work *and* data communication (round trip time) ($T_{j,g}$), given in Eq. (1):

1 $$\begin{aligned} T_{G} = \sum _{j=1}^{4}{max(T_{j,c}, T_{j,g})} \end{aligned}$$

### Experimental setup

We used five custom experimental MS datasets (denoted $E_{i}$) of increasing size for our experimentation. These datasets were constructed by unionizing/appending several Pride Archive (PXDxxxxx) datasets. The details of the five datasets are as follows: $E_{1}$: PXD009072, $E_{2}$: PXD020590, $E_{3}$: PXD009072 $\cup$ PXD010023 $\cup$ PXD012463 $\cup$ PXD013074 $\cup$ PXD013332 $\cup$ PXD014802 $\cup$ PXD015391, $E_{4}$: PXD015890, and $E_{5}$: PXD009072 $\cup$ PXD020590 $\cup$ PXD013332 $\cup$ PXD015890. The search experiments were conducted against increasing size theoretical databases constructed by incrementally adding combinations of commonly occurring post-translational modifications (PTMs) to the UniProt Homo sapiens (UP000005640) proteome sequence database. The database was fully digested in-silico using Trypsin with the Digestor tool^29^ using the following settings: allowed missed cleavages: 2, peptide lengths: 6 to 46, and peptide masses: 500 to 5000Da. The theoretical spectra were simulated by generating b- and y-ions for up to +3 charge, and no isotope error and decoys. Cysteine carbamidomethylation was set as fixed modification for all experiments whereas up to 5 PTMs per peptide were chosen from the combinations of methionine oxidation, arginine and glutamine deamidation, serine, threonine and tyrosine phosphorylation. The closed-search criterion in our experiments was set to $\delta M \approx \pm$ 1 Da instead of a few ppms to cover the differences in monoisotopic or average masses, and rounding off errors across tools. The open-search criteria was set between ±100 to 500 Da across experiments. The fragment-ion mass tolerance ($\delta F$) was set to ±0.01 Da for all tools (where applicable) and experiments. All experimental MS datasets $E_{i}$ were converted to the MS2 format using the MSConvert tool^30^. The experimental MS data pre-processing settings were set to minimal across tools for nearly identical algorithmic work (fairness), and were set as follows: precursor masses: 500 to 5000Da, precursor charges: +1 to +4, minimum shared fragment-ions for candidacy: 4, minimum candidate hits for statistical scoring: 4, de-noising: pick only top 100 or 150 peaks by intensity, no data reconstruction, no mass calibrations, no precursor peak removal, and no n-term methionine clipping.

*Runtime environment* All experiments were run on the Dragon scientific computing cluster at the Florida International University (FIU). The cluster is equipped with 6 compute nodes each powered by 2 $\times$ Intel Xeon Gold 5215 (2 $\times$ 10 cores), 2 $\times$ NVIDIA RTX A6000 GPU (84 SMs, 48GB DRAM), 2 NUMA nodes $\times$ 128GB DRAM, 3TB SSD local storage. The compute nodes are interconnected with each other and the storage nodes (18TB) via a 10Gbps Ethernet interconnect in star topology.

### Correctness analysis

**Figure 1:**
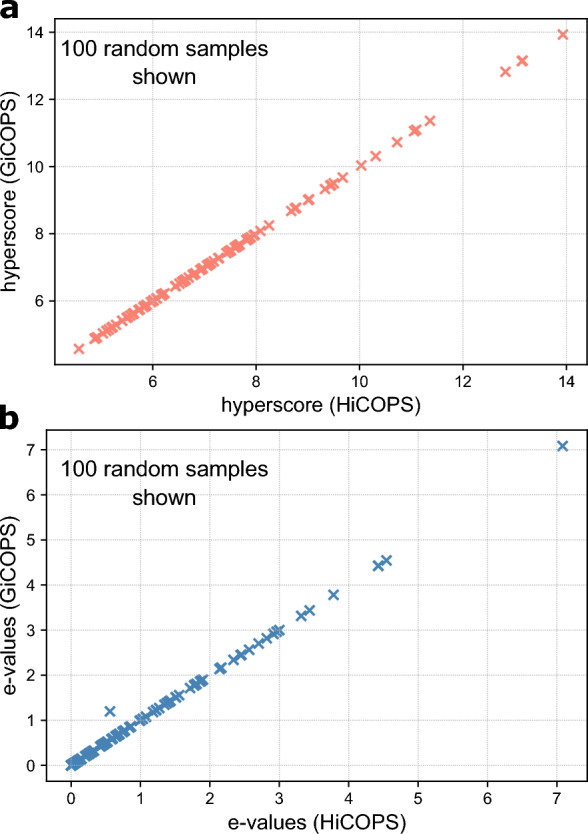
Correctness analysis. (**a**,**b**) Comparison of 100 samples out of 208K data points of the GiCOPS- (y-axis) and HiCOPS-computed (x-axis) hyperscores (**a**) and expected scores (or e-values) (**b**) is shown. The results show near 100% consistency between the computed results across all 208K data points, but were sampled to 100 for plotting feasibility.

We measured the correctness of GiCOPS’s algorithms by comparing the results, i.e., peptide identifications, hyperscores and expected-scores, computed by GiCOPS against its CPU-only HPC framework, HiCOPS, for single- and multi-node runs. The experiments were performed by searching all experimental MS-datasets against various database combinations in open and closed search modes. Figure 1a and b show a comparison of scores (100 samples out of 208K) computed by GiCOPS and HiCOPS when searching the experimental MS dataset $E_{1}$ against the Homo sapiens database with methionine oxidation as PTM in both search modes. The results from either mode were combined by concatenating them and removing the duplicate results.

Figure 1a and b depict that both HiCOPS and GiCOPS compute identical and consistent scores and peptide identifications of the experimental spectra regardless of the number of parallel nodes. We also observed a small number ($\ge$0.05%) of discrepancies in the GiCOPS’s computed scores across runs. Upon investigation, we found that this stems from the reduction loops (when multiple reduced candidate peptides have equal scores) and floating-point precision errors (when database candidate peptides lie just at the $\delta M$ boundaries). We mitigated the first problem by modifying the reduction loops to pick the peptides with the smaller index. We mitigated the second problem by slightly increasing the $\delta M$ boundaries for GiCOPS experiments and did not convert floating point computations to integers like in HiCOPS to avoid a 50% performance loss in database indexing step for our GPUs (see Performance Evaluation section). Finally, we compared the peptide identification results between GiCOPS and MSFragger^7^ (a database search engine that employs a similar, but proprietary, fragment-ion based algorithm) and the obtained correlation between the two tools was identical to the correlation discussed for HiCOPS and MSFragger in Ref.^4^. This is as expected because GiCOPS is only the GPU-accelerated version of the HiCOPS’ algorithmic kernels.

### Speed comparison against HiCOPS

**Figure 2:**
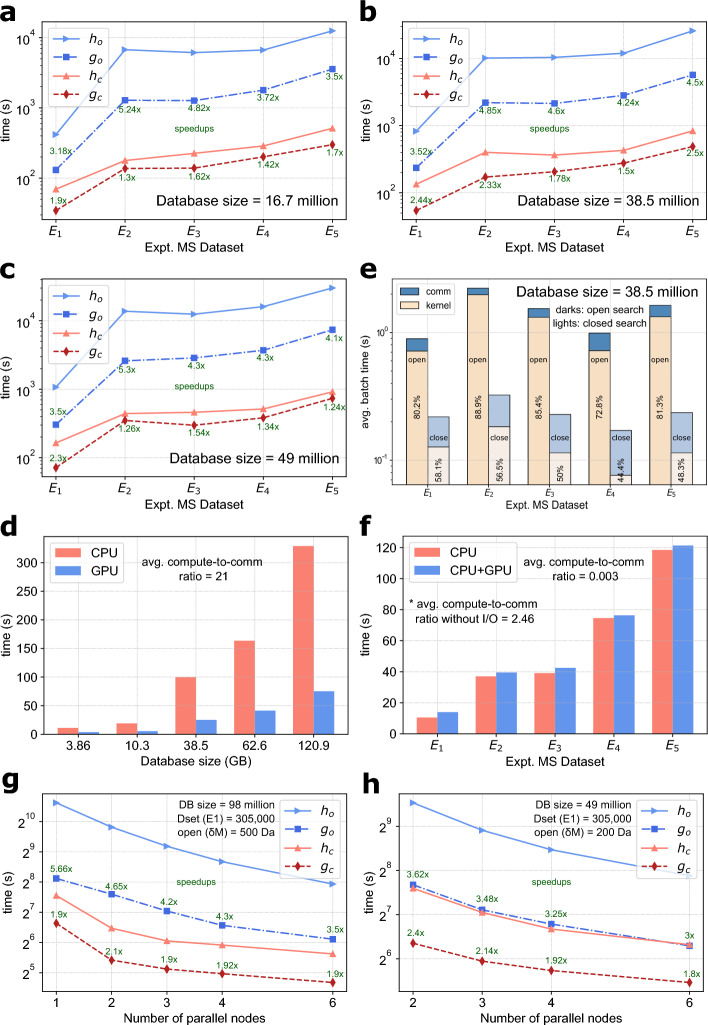
Speed Comparison with HiCOPS. (**a**) to (**c**) GiCOPS provides on average, a 3-5$\times$ and 1.2-2$\times$ speedup over HiCOPS in open (blue; $g_o$ and $h_o$) and closed (reds; $g_c$ and $h_c$) search modes respectively across experiments (labeled in green). (**d**) The percentage database search kernel time for the experiments in figure (**b**) drops from 70-90% (dark colored bars) in open-search to 45-55% (light colored bars) in closed-search significantly depreciating its speedup. (**e**,**f**) GiCOPS exhibits 3-6$\times$ speedup and no speedup compared to HiCOPS for the database construction and experimental MS data pre-processing steps respectively due to their corresponding computation-to-communication ratios. (**g**,**h**) Similar overall speedup results are seen for multi-node GiCOPS and HiCOPS runs for both search modes.

We measured the speed improvement provided by GiCOPS against its CPU-only version, HiCOPS, by searching all five datasets ($E_{i}$) against increasing size theoretical databases constructed by adding common PTMs in the Homo sapiens database in both open- (at $\delta M$ 500 Da) and closed-search (at $\delta M$ = 1 Da) modes. Our experimental results in Fig. 2a to c show that GiCOPS’s speedup (shown as $g_{o}$ and $g_{c}$ for open and closed search respectively, labeled in green) over HiCOPS (shown as $h_{o}$ and $h_{c}$ for open and closed search respectively, labeled in green) varies between 3 to 5$\times$ in the open-search setting and between 1.2 to 2$\times$ in closed-search setting respectively. The results also show that the achieved speedups are proportional to the computational workload, i.e., database size, dataset size, and search setting, across experiments. In particular, the computational workload of an experiment is quadratically proportional to the search mode (open or closed) setting as it controls the database lookup constraints for each given experimental spectrum. In other words, it controls the total number of pairwise comparisons to be computed between the experimental spectra in the dataset and the theoretical spectra in the database, for a given experiment. In contrast, the computational workload varies linearly with the database and experimental dataset sizes. Note that the computational workload also varies with several other factors such as the properties and nature of the data. e.g., origin species of data, signal-to-noise ratio. This is in-line with our performance analysis discussion in the Performance analysis section (see Eq. (5)).

The search mode also directly affects the computation-to-communication ratio of the database search step on an experiment which makes up a significant fraction of GiCOPS’s overall speedup. Figure 2d plots the computation-to-communication ratios of the database search superstep of the experiments in Fig. 2b. Comparing the two figures, we can see that the computation-to-communication ratio drops from $\sim 85$% kernel time (or 5.66 = 85/15 ratio) in the open-search mode to $\sim 50$% kernel time (or 1 = 50/50 ratio) in closed-search mode impacting the corresponding speedups. For instance, Fig. 2b and d show that the experiment searching the dataset $E_2$ exhibits speedups of 4.85 and 2.33 corresponding to the percentage (database search) kernel times of 89% and 56.5% in the open- and closed-search modes respectively. The same trend is observed for all experiments in Fig. 2a and  c as well. Therefore, we can infer that the experiments with similar computation-to-communication ratios also roughly exhibit similar speedups unless data sizes are highly dissimilar.

The effect of the computation-to-communication ratio to the achieved speedup is also particularly evident in the speedup results for the first two GiCOPS’s supersteps in Fig. 2e and f respectively. Here we observe, on average, computation to communication ratios of 21 (95.45% kernel time) and 0.003 (0.29% kernel time) for the database construction and experimental data pre-processing steps respectively resulting in corresponding speedups. Note that the I/O constitutes more than 90% of the total time in the data preprocessing step for most experiments. However, as noted in Methods section, experimental datasets only require preprocessing only once and is skipped in subsequent experimental runs. Further, since the database construction consistently depicts large speedups and only depends on the database size, it does have a significant positive impact on the overall speedup of small-scale experiments. i.e., closed-search experiments, experiments with small dataset sizes such as $E_1$ This impact can be particularly seen for $E_1$ in closed-search mode in Fig. 2c. We also measured the performance of GiCOPS against HiCOPS on distributed-memory architecture by searching the dataset $E_1$ against larger database sizes, on our distributed-memory HPC cluster. The experimental results in Fig. 2g and h depict a similar trend in the GiCOPS’s speedup for either search modes relative to the computational workload per node (Amdahl’s Law).

### Speed comparison against existing GPU methods

We attempted to measure GiCOPS against several existing GPU-based database peptide search software for speed comparison. These tools included Tempest, Tide-for-PTM-search, GPUScorer, ProteinByGPU, MIC-Tandem, and PaSER. We could only perform experiments using Tide-for-PTM-search, referred to as *GPU-Tide* in the rest of the text, as the other software tools were either outdated, unavailable, incompatible, proprietary, or faulty (Supplementary Section 3). Therefore, we compared GiCOPS to the GPU-Tide across three experiments in both closed- (at $\delta M$ = 1 Da) and open-search modes (at $\delta M$ = 100 Da). The $\delta M$ was reduced to 100 Da from 500 Da in other experiments, due to GPU-Tide software limitations.

**Figure 3:**
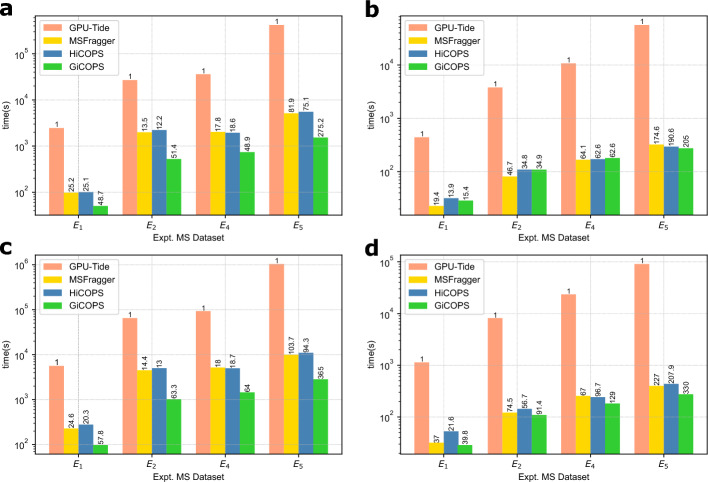
Speed Comparison with GPU-Tide. (**a**,**b**) First experiment set: The execution time results (in $\log _{10}$ scale) depict that all MSFragger, HiCOPS, and GiCOPS outperform GPU-Tide by $\ge 10\times$ (achieved speedups over GPU-Tide written on top of bars) in both open (**a**) and closed (**b**) search modes. This speedup increases to $\ge 50\times$ for larger experiments. (**c**,**d**) Second experiment set: Similar results are seen in both open (**c**) and closed (**d**) search modes. Note that the open-search setting in these two experiment sets was set to $\delta M$ = 100 Da instead of $\delta M$ = 500 Da in other experiments due to $\delta M$ limit of 100 Da in GPU-Tide.

In the first set of experiments, we searched all our datasets: $E_{1}$ to $E_{5}$, except $E_{3}$ (for which, GPU-Tide crashed) against the Homo sapiens database using only methionine oxidation as PTM (size: 3.89 million) in both modes. In the second experiment, we also added the arginine and glutamine deamidation as PTMs to the database (size: 10.3 million) and searched all above datasets in both modes. The obtained wall time results in Fig. 3a and b depict that GiCOPS outperforms GPU-Tide by more than 10$\times$ ($>50\times$ for larger experiments) in the first experiment set in both open- (Fig 3a) and closed-search (Fig 3b) modes. Similar results are seen for the second experiment set in Figures 3c (open) and 3d (closed). To explain the speedup results, we also ran the same experiments using HiCOPS and MSFragger and the results in Fig. 3a to  d show that both the CPU-only tools also outperform the GPU-Tide by $>10\times$. This is primarily because the GPU-Tide only relies on speeding up the spectral dot product (hyperscore) computations and does not leverage (or accelerate) any database filtration techniques (reductive optimizations) commonly employed in modern CPU-based open-search search algorithms^4,6,7,24^ leading to this performance gap. Other existing GPU-based database search algorithms also do not leverage any filtration techniques as discussed in Supplementary Section 2. Notice that the GiCOPS achieves slightly less speedups over HiCOPS in these experiments as compared to results in Fig. 2 due to the reduced $\delta M$ setting. Finally, the execution times for GPU-Tide span several hours to multiple days even for our relatively small experiments, making it unfeasible to run larger experiments with it.

### Performance evaluation

We analyzed the GiCOPS’s GPU throughput using the Instruction Roofline Model^27^. The Instruction Roofline Model is an intuitive visual method to understand and evaluate the performance and bottlenecks of integer-operation-intensive kernels relative to the theoretical maximum performance^27^. Since more than 85% of the GiCOPS’s runtime consists of the database search step for most real-world experiments, we focus our performance analysis and optimizations to that kernel only. For our performance analysis, we first computed the GPU’s theoretical maximum throughputs - i.e. the *roofs* in the model - were computed using the Empirical Roofline Toolkit^31^. We then ran a GiCOPS experiment searching a batch of 10000 experimental MS spectra against the Homo sapiens database incorporating methionine oxidation, arginine and glutamine deamidation, and serine, threonine and tyrosine phosphorylation as PTMs in open- and closed-search modes using the NVIDIA Nsight Compute (NCU). The collected metrics, along with the ERT parameters were then used to build the Instruction Roofline Model for the both search modes using the methods explained in^27^.

**Figure 4:**
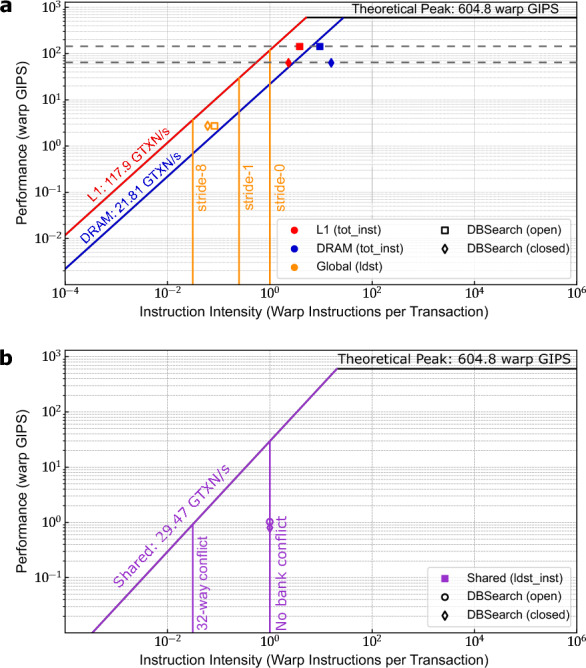
Instruction roofline model. (**a**) GiCOPS’s database search kernel exhibits a throughput of 143.6 and 64.6 warp GIPS in open- (squares) and closed- (diamonds) search modes respectively on NVIDIA RTX A6000 GPU. The achieved performances correspond to 48% and 21.5% of the theoretical integer-instruction peak as well as >99% corresponding thread-predication peaks (dotted black lines). All GPU peak throughputs were measured using the ERT toolkit^31^. (**b**) The shared memory performance of GiCOPS is near-ideal with negligible number of bank conflicts affecting the performance.

The Instruction Roofline Model shown in Fig. 4a depicts that the GiCOPS’s database search kernel in open (shown as squares) and closed-search (shown as diamonds) achieves a throughput - in warp Giga Instructions per Second (warp GIPS)^27^ - of 143.608 warp GIPS and 64.642 warp GIPS respectively. This achieved throughput corresponds to 48% and 21.5% of theoretical integer instruction peak on NVIDIA RTX A6000 as almost all computational instructions in GiCOPS are integer-based. The integer instruction peak performance is computed by scaling the GPU’ theoretical peak instruction throughput (604.8 warp GIPS) by the ratio of number of INT cores per warp to the warp size (=16/32 for NVIDIA RTX A6000 (Ampere architecture)) resulting in $0.5\times 604.8 = 302.4$ warp GIPS. Also, note that the LDST instructions make up a significant fraction of GiCOPS’s instruction mix which further affects its maximum theoretical achievable throughput. Figure 4a also shows that the effect of thread predication (branching) negligible as the achieved performance (squares and diamonds) lie right on the dotted black lines that correspond to the thread-predication-free respective throughputs of 145.67 warp GIPS (open-search) and 65.83 warp GIPS (closed-search). Finally, the Fig. 4a shows the DRAM (global memory) access pattern between stride-1 and stride-8 walls (vertical orange lines) indicating the expected intense usage of a 12-byte data structure to compute peptide similarity scores. Figure 4b depicts a virtually bank-conflict-free shared-memory performance of GiCOPS’s database search for both open- (square) and closed-search (diamond) modes.

## Discussion

In this paper, we presented GiCOPS framework which is a GPU-accelerated technique applied to traditional scientific computing database peptide search algorithms and the corresponding MS data flows. In stark contrast to several existing (SIMD-based) methods, the GiCOPS framework is designed for high-level SIMT-architectures, i.e. the implemented CUDA-based kernels can be directly deployed (with some fine-tuning) on most NVIDIA GPU, as well as ported/translated for AMD and Intel GPUs using HIP/ROCm, and SYCL^32^. We expect wide usage and experimentation and have made the GiCOPS code base publicly available for domain scientists and researchers to use. Our extensive experimentation using real-worl experimental datasets and databases reveals that GiCOPS exhibits speedups between 1.2 to 2$\times$ in closed-search and 3 to 5$\times$ in open-search mode, as compared to its CPU-parallel version (HiCOPS). GiCOPS also exhibits >10$\times$ speedup ($>50\times$ for larger experiments) as compared to the existing GPU-based methods in either search mode. The speed benefits provided by GiCOPS are especially significant for the compute-intensive (open-search) experiments.

Our proposed framework show that GPU based methods for database search-algorithms can significantly improve the overall performance of the workflows - improving both the throughput and execution times for pipelines that require executing large data sets e.g. meta-proteomics. While significant speedup results were obtained, the peptide identification accuracy achieved by GiCOPS depends on the underlying algorithmic pipelines being executed - and new algorithms that can improve the peptide deduction accuracy are not within the scope of this paper. Recent advances in the machine and deep learning based algorithms for the database peptide search have led to more accurate and confident peptide identifications (e.g. yHydra^33^). These complex models require massive amounts of computational resources and with the existing software infrastructure, require several hours and weeks to train, test and deploy^33,34^ these methods. Our future efforts of expanding and integrating the presented infrastructure into a ML/DL based HPC-accelerated database peptide search software will increase the speed at which models can be trained and tested - leading to better peptide deduction engines in the long run. We are confident that our proposed framework will help bridge the gap between the computational demands of current and future algorithms and machine-learning models and the available software infrastructure will meet these demands using our framework on clusters, and supercomputing machines. Our current and future research efforts will help accelerate and advance the scientific investigations in this application domain - and will act as a the fourth pillar for scientific investigation in the field of MS based omics including proteomics, and meta-proteomics.

## Methods

### Notations and symbols

For the rest of the manuscript, we will denote the size of the indexed theoretical spectra database as (*D*), a set of experimental MS data as ($Q=q_1,q_2,\cdots$) containing (*q*) spectra, with average length of ($\eta$) split across (*b*) batches, the CPU/GPU thread ids as (*tid*), GPU thread block size as ($\psi$), the CPU-GPU communication latency as ($\omega$) and bandwidth as ($\pi$). Note that the indexed theoretical spectra database will be referred to simply as the database in the rest of the paper.

### CPU-GPU pipeline

GiCOPS’s CPU-GPU pipeline consists of a global work queue *wq*, a priority queue *pq* and an oversubscribed scheduling thread $t_{s}$. In each GiCOPS’s step, the computational workload is divided across smaller chunks or files (either database or experimental data) which need to be processed by either CPU or GPU. Based on the computational algorithm and the amount of data, the priorities of CPU and GPU can be adjusted to favor one resource over the other as needed. The scheduling thread $t_{s}$ operates as a producer-consumer router and schedules each work unit in *wq* to CPU or GPU. The routing is done by moving the work units from the global queue to CPU or GPU’s local queues and thread signaling via C++ condition variables. When all compute resources are busy, the $t_{s}$ simply remains in the sleep state. A schematic of the CPU-GPU pipeline is shown in Supplementary Fig. 1 and the scheduling algorithm is illustrated in Supplementary Algorithm 1. Notice that this scheduling thread $t_{s}$ is different than the thread manager in HiCOPS which manages the speed of producer and consumer threads and is also employed in GiCOPS with additional functionality for GPU.

### Step 1: Database construction

**Figure 5:**
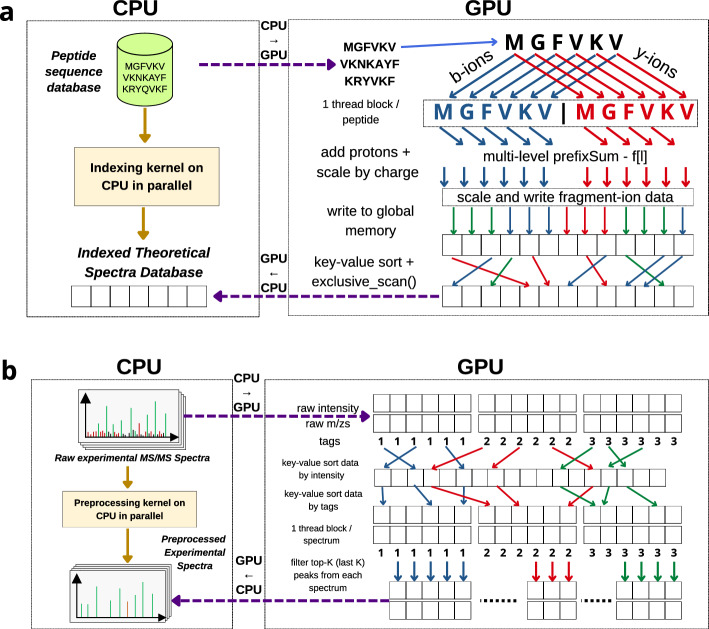
GiCOPS Steps 1 and 2. (**a**) Database Indexing: The CPU communicates the (small) peptide sequence database to the GPU where the theoretical MS-spectra data are generated and indexed using the CFIR-Index data structure. The CFIR-Index is then communicated back to the CPU. (**b**) Experimental MS-Data Preprocessing: The experimental MS data are streamed to the GPU side where they are (in current implementation) denoised using the Sorted Tag Array coupled peak picking algorithm. The preprocessed data are streamed back to the CPU.

In this step, GiCOPS constructs a database of indexed theoretical MS spectra data from one or more peptide sequence database(s) using the CFIR-Index^11^ data structure (Fig. 5a). At the CPU side, the peptide sequences and their PTM variants are generated and grouped by length. The peptide groups are communicated to the GPU where an instance of the CFIR-Index is computed for each group^11^. The peptide index is first computed using a two-fold parallel algorithm that computes the peptide precursor masses of each sequence and then Radix sorts them using their computed masses as *keys*. Here, each thread block of size *l* processes a peptide sequence of length $l+1$ and each thread processes an amino acid character. Then, another two-fold parallel kernel is then launched to generate and index the theoretical MS spectra data. Here each thread block is of size $s\times l$ where *s* is the number of ion-series to be generated. Typically, only b- and y-ions are generated so the *s* is 2. Inside each block, the first $tid < sl/2$ threads compute the forward ion-series (a, b, c) and operate on the amino acid character at $tid \mod l$, whereas the last $tid \ge sl/2$ threads compute the reverse ion-series (x, y, z) and operate on the amino acid character at $l - (tid \mod l)$. The threads write the computed amino acid masses at the index *tid* of a shared memory array (*S*). A multi-level (warp- and block-level) reduction algorithm is used to compute the prefix sum of *S*. Then, each thread $tid > l$ removes the sum of previous ion-series to get the current ion-series’s prefix sum by computing $S[tid] \leftarrow S[tid] - S[l * (tid / l)]$. The generated data are then scaled for ion-charges and written to the global memory. Once all theoretical MS spectra data are generated, the CFIR-Index’s fragment-ion index is computed using *stable sort-by-key*, and *lowerbound* kernels^35^. The GPU algorithm for fragment-ion construction is illustrated in Fig. 5a and Supplementary Algorithm 2. Finally, the constructed CFIR-Index instance is communicated to the CPU memory.

#### Remark

Since database indexing is a compute-intensive step, it can be entirely executed on the GPU-side as well with little to no aid from the CPU side in the CPU-GPU pipeline without significantly affecting the overall performance.

### Step 2: Experimental MS data preprocessing

In this step, GiCOPS pre-processes (i.e., normalize, clean, and filter) the experimental MS data and writes it back to the disk (Fig. 5b). At the CPU side, the experimental spectra are read in batches *Q* and streamed to the GPU. At the GPU side, the spectra in each batch are pre-processed and communicated back to the CPU side for indexing and writing back to the storage. For pre-processing, an algorithm like MSFragger and HiCOPS is employed where the top-K (*k* = 100-150 usually) data points are normalized and extracted from each spectrum. The spectra are first sorted using a *Sorted Tag Approach*^36^ and then the last or first-K data points depending on the sort-type are normalized and extracted. In the Sorted Tag Approach, all spectra arrays $q_{i}$ in a batch are concatenated to a global array $Q = q_{1},q_{2},..,q_{q}$. The global array *Q* is then sorted and then rearranged to the sorted versions of the spectra arrays $q_{i, sorted}$. To preserve the position information during the global sort, a *tag-array* (*T*) is initialized as $T[j] = q_{i}$ where $q_{i}$ is the spectrum id of the *j*-th data point in *Q*. For each wrangling operation on one or more dimensions of *Q*, the wrangling position information is stored in *I* which is then used to *gather* the remaining dimensions of *Q* as well as the *T*. Once all operations are done, Thrust’s *stable-sort-by-key* kernel^35^ is employed using *T* as key to gather the processed versions of spectra $Q = q_{1, sorted},q_{2, sorted},..,q_{q, sorted}$ in the correct order. Once sorted, another GPU kernel is launched where each sorted $q_{i,sorted}$ is processed by a thread block of size *K* where each thread normalizes and extracts the $q-tid$-th data point in $q_{i,sorted}$. The extracted data are written to a DRAM array $Q'$ along with the metadata (including new spectrum lengths) and communicated back to the CPU. The GPU algorithm for experimental MS data pre-processing is illustrated in Fig. 5b and the Supplementary Algorithm 3. An example of the Sorted Tag Approach is shown in Supplementary Fig. 2.

#### Remark

Similar to HiCOPS, this step is only executed only once per experimental MS data set. Once an experimental MS dataset is pre-processed and indexed by GiCOPS, this step is skipped for all subsequent runs unless required due to a change in the setting. e.g., base intensities, normalization, number of data points, or the dataset index is outdated. Also, since the current experimental data pre-processing is primarily a communication-intensive step, it can be executed entirely at the CPU-side without significantly affecting the overall performance.

### Step 3: Database peptide search

**Figure 6:**
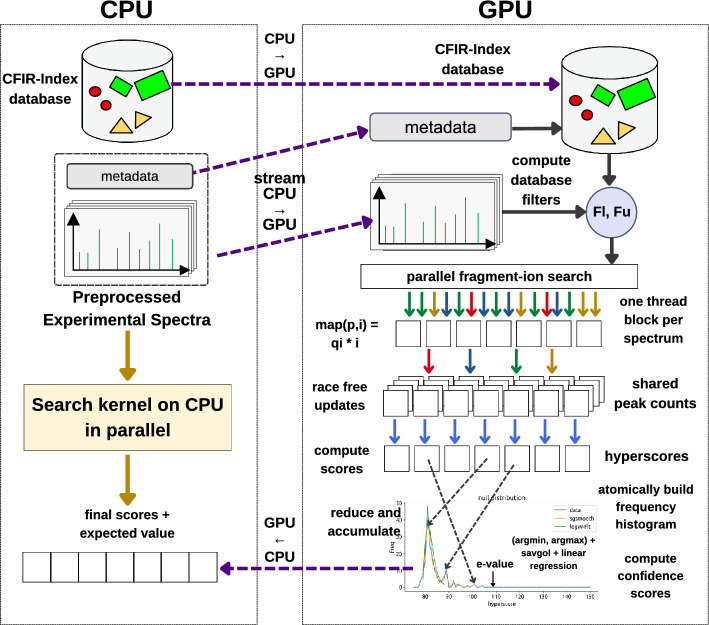
GiCOPS Steps 3 and 4. Database Peptide Search: The CFIR-Index and the pre-processed experimental MS data are communicated to the GPU in at once and stream fashion respectively. The GPU computes database filters $F_{lower}$ and $F_{upper}$ for each batch and using that computes the (map-reduce) fragment-ion match coupled hyperscore based database peptide search of Ref.^4^. Result Postprocessing: For single node case, the computed scores and their distributions from the last step are directly used to compute confidence scores which, along with peptide identifications are communicated back to the CPU. In multi-node case, the scores are first assembled globally as explained in Ref.^4^ before computing confidence scores.

This is the core computational step in the entire GiCOPS pipeline contributing over 90% of the total algorithmic workload in most real world experiments. In this step, GiCOPS searches the pre-processed experimental MS spectra data against the database using the fragment-ion search method also employed in Refs.^4,7,24^ (Fig. 6). Before the search, the CPU communicates all instance of the CFIR-Index to the GPU’s main memory. During the search, the CPU reads the batches of the pre-processed experimental MS data ($q \in Q$) from the storage or file system and streams them to the GPU. At the GPU side, these batches *Q* are searched against the indexed database (CFIR-Index) *D* and the results (top database matches *h* and null distribution of scores *N*) are communicated back to the CPU. The search kernel is launched in sub-batches of $\Lambda$ thread blocks depending on the GPU’s DRAM (default: $\Lambda =512$). Each thread blocks searches a spectrum *q* against the *D* using $\psi$ threads (128 (closed-search) $\ge \psi \ge$ 512 (open-search)) depending on the search mode. During the search, the peptide and fragment-ion mass filters are first computed (*lower-* and *upper-bounds*) using $\psi$-ary reduction trees and stored in shared-memory arrays $F_{lower}$ and $F_{upper}$. The fragment-ion search is then computed in data parallel fashion for each ion $i \in q \in Q$ between $F_{lower}[i]$ and $F_{upper}[i]$ and updated to a scorecard memory in DRAM. The massively GPU-parallel fragment-ion search is prone to race conditions, for which a two-step algorithm is employed (explained in Section 4.8.1) to alleviate the race conditions at a $\Theta (\log (\psi ))$ cost. Once all fragment-ions have been processed, the scorecard is data-parallel processed to compute the hyperscores *h*[*q*] and atomically build the null distributions *N*[*q*]. The number of candidate peptide-to-spectrum matches (database hits) processed and the maximum hyperscore $h_{max}[q]$ are computed in local per-thread variables during data-parallel execution and gathered at the end using optimized thread-shuffle based warp-wise block reductions (see Section 4.8.3). The GPU database peptide search kernel along with relevant reduction blocks are illustrated in Fig. 6 and Supplementary Algorithm 4. The final results (*h*[*q*] and *N*[*q*] ; $\forall q \in Q$) are communicated back to the CPU where they are encoded and written to the file system for distributed system-wide gathering, assembly and postprocessing^4^.

#### Remark

The database search step poses a complex mixed (compute- and memory-intensive) workload and constitutes a large fraction of the total execution time, it is imperative to focus the optimization and fine-tuning efforts to this kernel to yield the maximum performance. For that, we used the Nvidia Nsight Compute (NCU) tool to iteratively profile and analyze the performance hotspots, and implemented optimizations to minimize them (discussed in later sections).

### Step 4: Result postprocessing

In this step, the database peptide search results are processed to compute the confidence scores (expected values *ev*) of the peptide identifications (Fig. 6—confidence score computations). The CPU-GPU pipeline for this step depends on whether the GiCOPS is running on a single-node or (distributed) multi-node machine. In case of distributed memory case, all local results from the database search step for all experimental spectra batches *Q* are partitioned across the parallel nodes where they are de-serialized and assembled using signal shift and add operations at the CPU side as explained in HiCOPS^4^. The assembled batches of global results are communicated to the GPU DRAM. However, in the single-node case, the results are not communicated to the CPU at the end of database search step and the postprocessing step is fused into the database peptide search step. In either case, at the GPU side, the post-processing kernel is launched where each thread blocks computes the confidence scores for one experimental spectrum using its computed hyperscores *h*[*q*] and the null distributions *N*[*q*] from the database peptide search step. The GPU block size ($\psi$) is set to the maximum length null distribution histogram in the batch, computed as $\psi \leftarrow max(h_{max}[q])$. The GPU kernel implements vectorized versions of the Linear Tail Fit^37^ and Gumbel-Fit^4^ algorithms to compute expected values, built using thread-shuffle based block-wise reductions (see Section 4.8.1). The GPU kernel for the result postprocessing is illustrated in Supplementary Algorithm 5. The final results and confidence score are communicated to the CPU where they are either written to the file system or communicated to other nodes depending on the computing architecture (single or multiple-node computing) as explained in HiCOPS^4^.

#### Remark

The result post-processing step, although highly optimized due its compute-bound and GPU-friendly computational nature, contributes less than 1% of the total computational work in the database peptide search pipeline and thus, does not affect the overall performance yield.

### Performance analysis

The GPU-performance of the GiCOPS can be modeled by analyzing the performance of its individual steps as given in Eq. (1). In most real-world experiments, our experimental MS data will already be pre-processed meaning we can prune the experimental MS data pre-processing step (step 2) from our analysis. Further, we can prune the data post-processing step (step 4) from our analysis as it contributes to less than 1% of the total computations and does not affect the overall performance. Using this, we re-write Eq. (1) as:

$$\begin{aligned} T_{G} = max(T_{1,c}, T_{1,g}) + max(T_{3,c}, T_{3,g}) \end{aligned}$$

To analyze only the GPU-performance of GiCOPS, we assume that the CPU-GPU pipeline in GiCOPS only schedules the work units on the GPU resulting in:

$$\begin{aligned} T_{G} = T_{1,g} + T_{3,g} \end{aligned}$$

For simplicity, we can drop the ($_g$) subscript from the above equation resulting in:

$$\begin{aligned} T_G = T_1 + T_3 \end{aligned}$$

In the above equation $T_1$ corresponds to the time to communicate the peptide sequences to the GPU, construct the CFIR-Index and communicate the database index back to the CPU, whereas $T_3$ includes the time to communicate the database and experimental data batches to the GPU, compute the database search including computing mass tolerance filters, fragment-ion search and hyperscores, and finally communicate the results back to the CPU. Using the asymptotic time complexities of each algorithmic operation, and scaling factors $k_{i, j}$ where *i* denotes the step number and $k_{j}$ incorporates the number of parallel GPU cores, clock speeds, occupancy factor and the number of parallel thread blocks for the *j*-th algorithmic operation, we can write $T_1$ and $T_3$ as:

2 $$\begin{aligned} T_1 = k_{1,1}(D) + k_{1,2}(D \log D) + k_{1,3}(a\log (a)) + k_{1,4}(\omega + D/\pi ) \end{aligned}$$

and

3 $$\begin{aligned} \begin{aligned} T_3&= k_{3,1}(b\omega + (Q+D)/\pi ) + k_{3,2}(q \eta \log (\alpha )) + k_{3,3}(q \eta \sigma \log (\psi )) \\&\quad + k_{3,4}(q \mu \log (\psi )) + k_{3,5}(q\upsilon /\psi ) + k_{3,6}(b \omega + 2048q/\pi ) \end{aligned} \end{aligned}$$

In Eq. (2), *a* is the number of fragment-ion bins in the CFIR-Index whereas in Eq. (3), $\alpha$ is the average fragment-ion bin size in CFIR-Index, $\sigma$ is the average number of fragment-ion matches per experimental spectrum fragment, $\mu$ is the average number of candidate database peptides to be scored for an experimental spectrum, and $\upsilon$ is the average number of collisions in atomic construction of null distributions. Combining all above equations, we have:

4 $$\begin{aligned} \begin{aligned} T_G&= k_{1,1}(D) + k_{1,2}(D \log D) + k_{1,3}(a\log (a)) + k_{1,4}(\omega + D/\pi ) \\&\quad + k_{3,1}(b\omega + (Q+D)/\pi ) + k_{3,2}(q \eta \log (\alpha )) + k_{3,3}(q \eta \sigma \log (\psi )) \\&\quad + k_{3,4}(q \mu \log (\psi )) + k_{3,6}(b \omega + 2048q/\pi ) \end{aligned} \end{aligned}$$

Let us split the Eq. 4 into computational ($T_P$) and overhead ($T_O$) to analyze the effect of different parameters on both parts and the overall performance.

5 $$\begin{aligned} \begin{aligned} T_P&= k_{1,1}(D) + k_{1,2}(D \log D) + k_{1,3}(a\log (a)) + k_{3,2}(q \eta \log (\alpha )) \\&\quad + k_{3,3}(q \eta \sigma \log (\psi )) + k_{3,4}(q \mu \log (\psi )) \end{aligned} \end{aligned}$$

and

6 $$\begin{aligned} T_O = k_{1,4}(\omega + D/\pi ) + k_{3,1}(b\omega + (Q+D)/\pi ) + k_{3,5}(q\upsilon /\psi ) + k_{3,6}(b \omega + 2048q/\pi ) \end{aligned}$$

From Eqs. 5 and 6, it can be seen that increasing either the database size (*D*) or the experimental dataset size $Q=q\eta$ the $T_P$ dominates over $T_O$ and vice versa for smaller database and dataset sizes. However, the most impactful factors in the overall GiCOPS’s GPU-performance are the peptide ($\delta M$) and fragment-ion ($\delta F$) mass tolerances impacting the $\sigma$ and $\mu$ in equation 5. Since both these factors appear in quadratic terms of $T_P$, they can significantly boost or diminish the computational workload in $T_P$ even for large *D* and *Q*. For instance, for large *D* and *Q* but small $\delta M$ and $\delta F$, the $T_P$ would significantly drop to only database indexing factors ($k_{1,j}$) and the $T_O$ may dominate the overall performance.

#### Remark

GiCOPS provides a significant GPU-acceleration (up to 5 $\times$) for database search kernel in open-search application even for small to medium sized database and dataset sizes. In closed-search the communication overheads result in a performance drop even for large database and dataset sizes. In either search mode, the GPU-accelerated database indexing step is unimpacted and provides a reasonable speedup over CPU-only code.

### Optimizations

The following sections discuss the algorithms and optimization techniques employed to efficiently alleviate the race conditions and boost the achieved performance in GiCOPS.

#### Race conditions in fragment-ion matching

The fragment-ion matching kernel filters the number of database candidates for a given experimental spectrum by computing the number and nature of shared fragment-ions between the experimental spectrum and the theoretical spectra in the database. The matches are recorded using a scorecard data structure updating (fetch, update, write) which in a parallel algorithm design results in race conditions. Profiling the code reveals that fragment-ion matching constitutes more than 50% of the total computational workload (both CPU and GPU) in the database peptide search step making it the most important kernel to be optimized. In our CPU-only parallel design, race conditions are alleviated by modifying the granularity of parallelism which is not applicable for GPUs.

Therefore, in GiCOPS, we implement a two-step method to eliminate these possible race conditions at the cost of $\Theta (\log (\psi ))$ operations, for the $\psi$ threads to make a parallel update to the scorecard. The first step involves *stabilizing* the sort operations during the CFIR-Index construction in the database indexing step. Stable sort operations ensure that the indexed fragment-ions within a $\delta F$ originating from the same peptide id are placed at adjacent locations. This also ensures that the GPU threads that may participate in a race condition when updating fragment-ion match scores are also located adjacent to each other. We exploit this locality information to eliminate the race conditions by applying a block-wise reduction in $\log (\psi )$ clock cycles using the algorithm illustrated in Supplementary Section 4, before writing the reduced fragment-ion scores.

#### Performance tuning

We employed the Nvidia Nsight Compute (NCU) to iteratively collect and analyze several performance metrics for the database search kernel, and fine tune its performance by adjusting the thread grid size, reducing the shared memory and register usage, eliminating bank conflicts, reducing the required thread barriers, padding certain data structures, and interleaving compute and memory operations where possible. The overall result of performance tuning was a 25% boost to the overall throughput (incorporated in the reported results), speed of light performance (12.1% compute and 80.06% memory), occupancy factor (80% theoretical max), active blocks (79.6% theoretical max), and the shared memory bank conflicts ($<0.1\%$ transactions).

#### Optimized reductions

Database peptide search algorithms frequently execute memory lookup kernels including *max*, *min*, *argmax*, *argmin*, *blocksum*, *lowerbound*, *upperbound*. Therefore, it is important to optimize these and other reduction operations in GiCOPS. To do this, we implement these kernels in GiCOPS using reduction trees that leverage CUDA’s warp shuffle intrinsics for optimized and constant space reductions. The reductions are performed in a two-step reduction where each warp is first reduced using warp shuffles. Then, the reduced values from each warp are collected in a shared memory array of $\le 32$ elements. These elements are collected by the zeroth warp and reduced again to compute the final value. Furthermore, several kernels such as *max* and *argmax* can and are fused together and computed in the same reduction kernel when possible. Furthermore, the *search* operations are implemented as vectorized *k*-ary search tree where *k* is the thread block size. A generic block-wise reduction algorithm using warp shuffles is illustrated in Supplementary Algorithm 6.

#### Compile-time computations

The fragment-ion based database search algorithm in GiCOPS, computes the hyperscore similarity metric^38^ between millions of pairs (many-to-many) of theoretical and experimental MS spectra. Consider a pair of spectra $\nu$ and $\xi$, the number of shared b- and y-ions between them is $n_b$ and $n_y$ respectively with corresponding intensities $i_{b, j}$ and $i_{y, j}$, then the hyperscore similarity between them is given in equation 7. Notice that the first two terms in equation 7 compute $\log$ of factorial of $n_b$ and $n_y$ which can be pre-computed for $0 \ge n \ge 120$ at compile time to avoid repetitive *O*(*n*!) on-the-fly computations. To do this, we precompute a data structure at compile-time (Supplementary Fig. 4) and employ a dynamic programming algorithm computing and memoizing: $\log (n!) = \log (n) + \log ((n-1)!)$. The dynamic programming array is communicated to the GPU’s constant memory at initialization stage. Also, note that this algorithm also avoids 64 bit overflow when computing *n*! for $n \ge 21$.

7 $$\begin{aligned} hyperscore(\nu , \xi ) = \log (n_{b}!) + \log (n_{y}!) + \log (\sum _{j=1}^{n_{b}}{i_{b,j}}) + \log (\sum _{k=1}^{n_{y}}{i_{y,k}}) \end{aligned}$$

### Supplementary Information

Supplementary Information.

## Data Availability

All datasets used in this study are publicly available from the Pride Archive via: https://www.ebi.ac.uk/pride/archive/projects/<AN> where <AN> is the accession number for each dataset mentioned in the text. For example, the dataset with the accesssion number: PXD015384, can be accessed via the link: https://www.ebi.ac.uk/pride/archive/projects/PXD015384. The Homo sapiens proteome sequence database used in this study is publicly available from UniProt via: https://www.uniprot.org/proteomes/UP000005640.

## Code availability

The GiCOPS software has been implemented as a part of the HiCOPS software framework using object-oriented C++17, MPI, CUDA, OpenMP, Python, Bash and CMake. The GPU performance was instrumented with NVIDIA Nsight Compute (NCU). GiCOPS is under active development at the Parallel Computing and Data Science Laboratory (PCDS Lab) at the Florida International University. The source code is available at https://github.com/pcdslab/gicops. The Instruction Roofline metrics were collected and analyzed using an in-house software based on NCU and^39^, and is available at https://github.com/pcdslab/Instruction_roofline_scripts.

## References

[1] Eng JK, McCormack AL, Yates JR (1994). An approach to correlate tandem mass spectral data of peptides with amino acid sequences in a protein database. J. Am. Soc. Mass Spectrom..

[2] Craig R, Beavis RC (2003). A method for reducing the time required to match protein sequences with tandem mass spectra. Rapid Commun. Mass Spectrom..

[3] Nesvizhskii AI (2010). A survey of computational methods and error rate estimation procedures for peptide and protein identification in shotgun proteomics. J. Proteomics.

[4] Haseeb M, Saeed F (2021). High performance computing framework for tera-scale database search of mass spectrometry data. Nat. Comput. Sci..

[5] Nesvizhskii AI, Roos FF, Grossmann J, Vogelzang M, Eddes JS, Gruissem W, Baginsky S, Aebersold R (2006). Dynamic spectrum quality assessment and iterative computational analysis of shotgun proteomic data toward more efficient identification of post-translational modifications, sequence polymorphisms, and novel peptides. Mol. Cell. Proteomics.

[6] Chi H, Liu C, Yang H, Zeng WF, Wu L, Zhou WJ, Niu XN, Ding YH, Zhang Y, Wang RM (2018). Open-pfind enables precise, comprehensive and rapid peptide identification in shotgun proteomics. bioRxiv.

[7] Kong AT, Leprevost FV, Avtonomov DM, Mellacheruvu D, Nesvizhskii AI (2017). Msfragger: Ultrafast and comprehensive peptide identification in mass spectrometry-based proteomics. Nat. Methods.

[8] Eng JK, Searle BC, Clauser KR, Tabb DL (2011). A face in the crowd: Recognizing peptides through database search. Mol. Cell. Proteomics.

[9] McIlwain S, Tamura K, Kertesz-Farkas A, Grant CE, Diament B, Frewen B, Howbert JJ, Hoopmann MR, Kall L, Eng JK (2014). Crux: Rapid open source protein tandem mass spectrometry analysis. J. Proteome Res..

[10] Xu TPSK, Park SK, Venable JD, Wohlschlegel JA, Diedrich JK, Cociorva D, Lu B, Liao L, Hewel J, Han X (2015). Prolucid: An improved sequest-like algorithm with enhanced sensitivity and specificity. J. Proteomics.

[11] Haseeb M, Saeed F, Haseeb M, Saeed F (2019). Efficient shared peak counting in database peptide search using compact data structure for fragment-ion index. 2019 IEEE International Conference on Bioinformatics and Biomedicine (BIBM).

[12] Madsen JR, Awan MG, Brunie H, Deslippe J, Gayatri R, Oliker L, Wang Y, Yang C, Williams S, Madsen JR, Awan MG (2020). Timemory: Modular performance analysis for hpc. International Conference on High Performance Computing.

[13] Stevens R, Ramprakash J, Messina P, Papka M, Riley K (2019). Aurora: Argonne’s Next-Generation Exascale Supercomputer.

[14] Awan MG, Deslippe J, Buluc A, Selvitopi O, Hofmeyr S, Oliker L, Yelick K (2020). Adept: A domain independent sequence alignment strategy for gpu architectures. BMC Bioinform..

[15] Block B, Virnau P, Preis T (2010). Multi-gpu accelerated multi-spin monte carlo simulations of the 2d ising model. Comput. Phys. Commun..

[16] Niemeyer KE, Sung C-J (2014). Recent progress and challenges in exploiting graphics processors in computational fluid dynamics. J. Supercomput..

[17] Li J, Ranka S, Sahni S. GPU matrix multiplication. *Multicore Computing: Algorithms, Architectures, and Applications*, 345, (2013).

[18] Milloy JA, Faherty BK, Gerber SA (2012). Tempest: Gpu-cpu computing for high-throughput database spectral matching. J. Proteome Res..

[19] Kim H, Han S, Um J-H, Park K (2018). Accelerating a cross-correlation score function to search modifications using a single gpu. BMC Bioinform..

[20] Li Y, Xia L, Chi H, Chu X. Accelerating mass spectrometry-based protein identification using gpus. *BMC Bioinform.*, (2014). 10.1186/1471-2105-15-121 PMC4049470 24773593

[21] Li Y, Chi H, Xia L, Chu X (2014). Accelerating the scoring module of mass spectrometry-based peptide identification using gpus. BMC Bioinform..

[22] Li Y, Chu X, Li Y, Chu X (2012). Speeding up scoring module of mass spectrometry based protein identification by GPU. 2012 IEEE 14th International Conference on High Performance Computing and Communication and 2012 IEEE 9th International Conference on Embedded Software and Systems.

[23] He P, Li K, He P, Li K (2015). Mic-tandem: Parallel x! tandem using mic on tandem mass spectrometry based proteomics data. 2015 15th IEEE/ACM International Symposium on Cluster, Cloud and Grid Computing.

[24] Beyter D, Lin MS, Yanbao Yu, Pieper R, Bafna V (2018). Proteostorm: An ultrafast metaproteomics database search framework. Cell Syst..

[25] Devabhaktuni A, Lin S, Zhang L, Swaminathan K, Gonzalez CG, Olsson N, Pearlman SM, Rawson K, Elias JE (2019). Taggraph reveals vast protein modification landscapes from large tandem mass spectrometry datasets. Nat. Biotechnol..

[26] Geer LY, Markey SP, Kowalak JA, Wagner L, Ming X, Maynard DM, Yang X, Shi W, Bryant SH (2004). Open mass spectrometry search algorithm. J. Proteome Res..

[27] Ding, N. & Williams, S. An instruction roofline model for gpus. In *2019 IEEE/ACM Performance Modeling, Benchmarking and Simulation of High Performance Computer Systems (PMBS)*, pp. 7–18, (2019).

[28] Tiskin A (2011). BSP (Bulk Synchronous Parallelism).

[29] Sturm M, Bertsch A, Gröpl C, Hildebrandt A, Hussong R, Lange E, Pfeifer N, Schulz-Trieglaff O, Zerck A, Reinert K (2008). Openms-an open-source software framework for mass spectrometry. BMC Bioinform..

[30] Adusumilli R, Mallick P, Adusumilli R, Mallick P (2017). Data conversion with proteowizard msconvert. Proteomics.

[31] Lo YJ, Williams S, Van Straalen B, Ligocki TJ, Cordery MJ, Wright NJ, Hall MW, Oliker L, Lo YJ, Williams S (2014). Roofline model toolkit: A practical tool for architectural and program analysis. International Workshop on Performance Modeling, Benchmarking and Simulation of High Performance Computer Systems.

[32] Haseeb M, Ding N, Deslippe J, Awan M, Haseeb M, Ding N, Deslippe J, Awan M (2021). Evaluating performance and portability of a core bioinformatics kernel on multiple vendor gpus. 2021 International Workshop on Performance, Portability and Productivity in HPC (P3HPC).

[33] Altenburg, T., Muth, T. & Renard, B.Y. yhydra: Deep learning enables an ultra fast open search by jointly embedding ms/ms spectra and peptides of mass spectrometry-based proteomics. *bioRxiv*, pp. 2021–12, (2021).

[34] Tariq MU, Saeed F (2021). Specollate: Deep cross-modal similarity network for mass spectrometry data based peptide deductions. PLoS ONE.

[35] Bell N, Hoberock J, Bell N, Hoberock J (2012). Thrust: A productivity-oriented library for cuda. GPU Computing Gems Jade Edition.

[36] Awan MG, Saeed F (2016). Ms-reduce: An ultrafast technique for reduction of big mass spectrometry data for high-throughput processing. Bioinformatics.

[37] Fenyö D, Beavis RC (2003). A method for assessing the statistical significance of mass spectrometry-based protein identifications using general scoring schemes. Anal. Chem..

[38] Craig R, Beavis RC (2004). Tandem: Matching proteins with tandem mass spectra. Bioinformatics.

[39] Ding N, Awan M, Williams S (2022). Instruction roofline: An insightful visual performance model for gpus. Concurr. Computat. Pract. Exp..

