# Supplementary material for: GPU-acceleration of the distributed-memory database peptide search of mass spectrometry data
Source: Sci Rep. 2023 Oct 31;13:18713. doi: 10.1038/s41598-023-43033-w (PMC10618243; doi:10.1038/s41598-023-43033-w)
Supplement: Supplementary file 1 — Supplementary Information. [file 41598_2023_43033_MOESM1_ESM.pdf]

Supplementary Text: GPU-Acceleration of the Distributed-Memory Database Peptide Search of Mass Spectrometry Data

Muhammad Haseeb[1], and Fahad Saeed[1,2,3,4]

[1] *Knight Foundation School of Computing and Information Sciences, Florida International University, Miami, FL, USA*
[2] *Biomolecular Sciences Institute (BSI), Florida International University, Miami, FL, USA*
[3] *Department of Human and Molecular Genetics, Florida International University, Miami, FL, USA*
Herbert Wertheim School of Medicine, Florida International University, Miami, FL, USA.
[4] *Corresponding Author. Email: fsaeed@fiu.edu*

October 20, 2023

1 Supplementary Sections

1.1 Supplementary Section 1

Related Work. Most early-age database peptide search algorithms employ complex matrix-vector and vector-vector operations such as Cross-Correlations (XCorr) [1], [2], Fast Fourier Transforms (FFTs) [3], [4], and Spectral (Vector) Dot Products (SDP) [5], [6], [7] to compute similarity scores between an experimental spectrum (or 2D vector) and a theoretical database spectrum. These computational motifs [8], as we know it, are efficiently parallelizable using SIMD and SIMT based hardware accelerators including vector engines, graphics processing units (GPUs) and Field Programmable Gate Arrays (FPGAs). Therefore, several research efforts focused on developing hardware-accelerated spectral similarity computing algorithms including GPU SDP and KSDP [9], FastPaSS [10] Tempest [11], ProteinByGPU [12], GPUScorer [13], Tide-for-PTM-search [14].. The common parallelization model employed in all above algorithms involves offloading the vector-vector similarity score computations by launching a thread block per computation. Most algorithms also employ optimization techniques including *vector sparsity* to efficiently exploit the compute, DRAM, memory bandwidth shared-memory resources [11], [9]. Several

algorithmic designs also explore further manual fine-tuning including loop unrolling, cache and register usage [12] to further improve the achieved throughput. Similarly, MIC-Tandem [15] accelerates the SDP computations using the (now discontinued) Intel Many Integrated Core (MIC) co-processors to achieve speedup. More recently, Bruker introduced PaSER which is a proprietary GPU-accelerated version of the ProLuCID-4D algorithm. As expected, most of the hardware-accelerated database peptide search algorithms report several orders-of-magnitude speedup over their respective CPU implementations. For instance, Tempest reports 8 to 13× speedups, Tide-for-PTM-search reports 2.7 to 5.8× speedups, and [9] reports 8 to 100× speedup for the SDP and KSDP kernels respectively.

1.2 Supplementary Section 2

Limitations in Existing GPU Algorithms. The parallelization methods employed in all existing GPU-accelerated database peptide search algorithms are based on the observation that the spectral similarity computations make up more than 80% or 90% of the total database peptide search computations [11], [9], [15], [10], [12]. While this is true for index-free (closed-search) database peptide search algorithms, modern CPU-based algorithms employ sophisticated data processing, machine learning and database filtering techniques [16], [17], [18], [19] to significantly reduce the number of required spectral similarity computations and achieve over 100× speedup over the existing GPU-based algorithms [16], [17], [20], especially for open-search.

Consequently, the computational profile has significantly shifted from compute-intensive (spectral similarity computation intensive) to data- and memory-intensive workflows [20]. For instance, MSFragger spends a significant fraction of its execution time in fragment-ion search involving memory lookups and updates. Similarly, TagGraph spends a significant fraction of their execution times in graph traversal-like computations to filter the potential database peptide candidates before computing scores. Consequently, the existing GPU-acceleration methods and optimizations based on matrix/vector computations cannot be employed to (optimally) accelerate the complex compute and memory-patterns of the modern database peptide search algorithms. Finally, the software/codebase of almost all existing GPU-accelerated algorithms are either unavailable, outdated, or unusable as explored in Supplementary Section 3.

1.3 Supplementary Section 3

Existing GPU Database Peptide Search Software. Tempest was obtained from its GitHub repository https://github.com/markadamo/tempest and built with GCC 9.4, SQLite 3.36, and CUDA 11.6. The experiments ran for days without any results. Debugging the code with GDB revealed that the program gets stuck in an infinite loop during OpenCL's GPU setup, no matter the input data or experimental settings. We tried to diagnose and fix, and even remove the problematic functions/loops altogether in the code (along with fixing other critical bugs) but were unsuccessful as the specific loop had multiple nested loop and compound `if-else` conditions with little documentation. We reported this issue to the Tempest developers at https://github.com/markadamo/tempest/issues/1 but it is still open as of writing this manuscript. ScoreByGPU was obtained from its website: http://www.comp.hkbu.edu.hk/~youli/ProteinByGPU.html but the software package contained only the pre-built binaries (no source code) for Windows OS built with CUDA 4.2 and so, could not be run on our experimental setup or any other computing resources available to us. We requested the authors for the source code or a Linux-based binary but did not get a response as of writing this manuscript. Tide-for-PTM-search was obtained from its GitHub repository at https://github.com/Tide-for-PTM-search/Tide-for-PTM-search and the pre-built binaries were run by installing the required libraries including CUDA 8.0, and MSToolkit. MCTandem was obtained from https://github.com/Logic09/MCtandem but could not be experimented with as it requires a discontinued and unavailable Intel Xeon Phi Coprocessor 7120P to run. PaSER is a commercial software based on a proprietary GPU-version of the ProLuCID algorithm developed by Bruker. We requested Bruker for a demo or an evaluation copy of PaSER but did not get a response as of writing this manuscript. Similarly, we could not find any binaries or source code for the GPU-based-SDP [9] algorithms (i.e., SDP and KSDP) so we reached out to the authors for a copy of the software but did not get a response as of writing this manuscript.

1.4 Supplementary Section 4

Eliminating Race Conditions in the GPU Fragment-Ion Search. Our algorithm for alleviating the race conditions in the GPU-accelerated fragment-ion search can be best explained using an example. Consider a simplified example where a GPU thread block of size ψ operate on an input data array A with ψ elements. The desired output is a score array B such that: $B[i] \leftarrow count(A/bin = i)$ where $bin \in \mathbb{N} - \{0\}$. On completion, each $B[i]$ will contain the count of elements in A with i as their tenth place digit. However, GPU-computing this kernel in a straight-forward manner as: $B[A[tid]/bin]+ = 1$ by all ψ threads may lead to race conditions. See the Supplementary Figure 3 for an example of this simplified problem.

To alleviate this, we leverage the fact that the A is pre-sorted, like the stable-sorted database index in CFIR-Index [21]. Processing a sorted A ensures that the threads updating the same $B[i]$ location will have adjacent thread ids, and can be identified by checking: $(A[tid]/bin = A[tid - 1]/bin)$. If this condition is true for a thread, then it belongs to a group of colliding threads and if it is false, then it is the leader of a thread group. Note that the threads not colliding with any other thread are also leaders of a group of size 1. Once the leaders and members are identified, we perform a block wise conditional reduction using the bin ids $(A[tid]/bin)$ as keys, updating the current bin scores (the sum of each group's size in this example) in each of the $\log(\psi)$ iterations. Once reduced, only the leader threads update the scorecard with their reduced values. The reduction algorithm is visually illustrated in the Supplementary Figure 3 as well as in Supplementary Algorithm 4 (lines 10 to 15).

Analysis. The best-case scenario for this algorithm would be when all ψ elements in A belong to the same group resulting in ψ collisions. The worst case scenario would be when none of the elements are colliding. In either case, using the above proposed algorithm will result in $\Theta(\log(\psi))$ operations to write ψ updates to the scorecard which is way more efficient than using *atomic* intrinsics leading to performance loss due to millions of such possible collisions.

2 Supplementary Figures

2.1 Supplementary Figure 1

CPU-GPU Pipeline Schematic. The CPU-GPU pipeline in GiCOPS consists of a scheduling thread t_s, a priority queue pq, a global work queue wq, and thread signaling semantics. The scheduling task repeatedly assigns all work items in the wq to the compute units in pq based on their priority and notifies them. The wq may be pre-filled, for instance, database or experimental data file handles, or may fill in parallel by data producer sub-tasks. The priorities maybe changed depending on the kind of the workload. For instance, assigning higher priority to the GPU or other accelerator units for compute-intensive workloads whereas assigning higher priority for memory- or communication-intensive workloads and so on. Supplementary Algorithm 1 illustrates the scheduling algorithm implemented by t_s

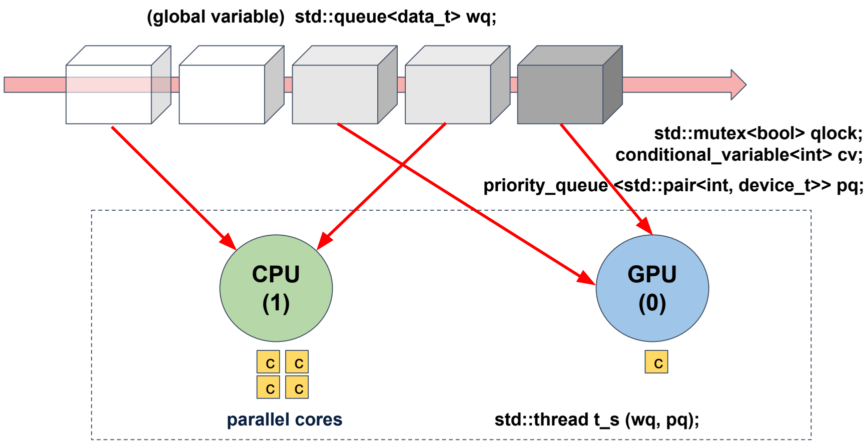

2.2 Supplementary Figure 2

Sorted Tag Approach (STA). Consider, we want to GPU sort three arrays a_i of sizes 2, 3, 4 respectively. Using the STA, we first flatten them into a global array $A = a_1, a_2, a_3$ and initialize a tag array $T = 1, 1, 2, 2, 2, 3, 3, 3, 3$. Then we first *StableSortByKey* using A as key and T as value, and then again using T as key and A as value. The resultant final array (A') now has the sorted versions of a'_i in the correct order.

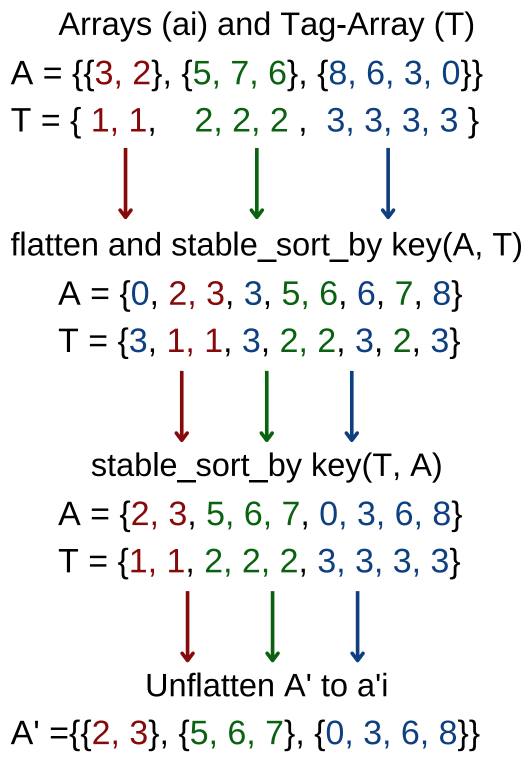

2.3 Supplementary Figure 3

Race Conditions in the GPU Fragment-Ion Search. Consider the input array A of size k, a GPU thread block of size $\psi = k = 11$, and a bin size of $bin = 10$, then computing the output array: $B[idx] = count(A[j]/bin = idx)$ in parallel by GPU threads would lead to race conditions. Our algorithm eliminates this by first ensuring that A is pre-sorted and then performing a block wide reduction before updating B. To do this, the threads first identify if they are the leaders of a bin-group and then using the bin numbers as keys ($key = A[tid]/bin$), the values (i.e. counts) are reduced. After reduction, only the leader threads update B with their final (reduced) values.

Input:

A = [2, 5, 10, 12, 18, 42, 65, 76, 78, 90, 98],

k = 11, bin = 10

Desired Output:

idx = 0, 1, 2, 3, 4, 5, 6, 7, 8, 9

B = [2, 3, 0, 0, 1, 0, 1, 2, 0, 2]

// initialized Keys and Vals

Keys = [0, 0, 1, 1, 1, 4, 6, 7, 7, 9, 9]

Vals = [1, 1, 1, 1, 1, 1, 1, 1, 1, 1, 1]

// add Vals only if same key

// Only write the colored (reduced) values to **B**

Vals = [1+1, 1, 1+1+1, 1+1, 1, 1, 1, 1+1, 1, 1+1, 1]

Algorithm

```
// shared arrays
__shared__ Keys[tid] = A[tid]/bin;
__shared__ Vals[tid] = 1; // initial count

bool isLeader = S[tid -1] != S[tid];

// reduction loop
for (i in range(0, log(k)))
        idx = tid + 2^i;
        if (Keys[idx] == key)
                Vals[tid] += Vals[idx];

if (isLeader)
      B[key] += Vals[tid];
```

2.4 Supplementary Figure 4

Compile-time computation of log(n!). The $\log(n!)$ for $0 \leq n \leq 121$ is computed at compile time and stored in a `constexpr` data structure called `lgfact`. The computations follow the dynamic programming equation: $\log(n!) = \log(n) + \log(n-1!)$ which also avoids 64-bit (`double`) overflow for $n \geq 21$. These values are communicated to the GPU's constant memory to be used during hyperscore computations.

```
// max shared b- or y-ions
const int maxshp = MAX_SHDPEAKS + 1; // 120 + 1

//
// computes log(x!) at compile time
//
template<int N>
struct lgfact {
    constexpr lgfact() : n() {

        n[0] = n[1] = log10(1);

        for (auto i = 2; i < N; i++) {
            n[i] = log10(i) + n[i-1];
        }
    }

    // needs to be const so it does not affect n
    auto operator[](int x) const { return n[x]; }

    // array n to store results
    double n[N];
};
```

3 Supplementary Algorithms

3.1 Supplementary Algorithm 1

Algorithm 1: GiCOPS's CPU-GPU Task Distribution Pipeline

Data: set of work units (W), min heap (pq) of compute units (u)
Result: work units scheduled on the CPUs and GPU

/* add GPU and CPUs to the pq with their priority as key */
$pq.push(0, gpu)$;
$pq.push(1, cpu)$;
/* assign all compute units to GPU */
/* or CPUs based on their priority */
for $w \in W$ **do**
    /* unique lock for condition variable */
    $unique\_lock()$;
    /* wait on a condition variable for a compute unit */
    $cv.wait(unique\_lock, !\,pq.empty())$;
    /* pop from the min heap */
    $u \leftarrow pq.pop()$;
    /* assign the current work unit w on u and notify */
    $assignNotify(w, u)$;
return;

3.2 Supplementary Algorithm 2

Algorithm 2: Step 1: GPU Fragment-Ion Index Construction

Data: peptide sequences (d) of length $l+1$, compute b- and y-ions only
Result: CFIR-Index's fragment-ion index (A, b)

```
/* compute my amino acid position */
p ← (tid mod l);
if tid > l then
    p ← l − p;
/* shared memory array S */
__shared__ S[];
/* write amino acid masses */
S[tid] ← AAmass(d[p]) + modMass(d[p]);
/* compute prefix sum of S by reduction */
for i ∈ 0 → ⌈log2(sl)⌉ do in parallel
    if tid ≥ 2^i then
        S[tid] ← S[tid] + S[tid − 2^i];
/* subtract the sum of b-ions and add H2O to y-ions */
if tid > l then
    S[tid] ← S[tid] + mass(H2O) − S[l];
/* compute protons and charged ions and write to global memory B */
for z ∈ maxZ do in parallel
    /* compute the new position in idx */
    idx ← (ls/2 − l) ∗ (tid > l) + z ∗ l + tid;
    /* write the computed data to B */
    B[idx] ← (z ∗ mass(H) + S[tid])/z;
/* A initialized to the indices of B */
for i ∈ size(A) do in parallel
    A[i] ← i;
/* stable sort A using B as keys */
StableSortByKey(B, A)
/* compute the lowerbounds b of each unique fragment-ion in A */
lowerbound(B, b);
return A, b;
```

3.3 Supplementary Algorithm 3

Algorithm 3: Step 2: GPU Expt. MS Data Preprocessing

Data: batch of experimental spectra (Q)

Result: Preprocessed batch of experimental spectra (Q')

```
/* compute splitters (tags)                                                  */
S ← exclusiveScan(Q);
/* initialize array tag array I                                              */
for q ∈ S do in parallel
    for j ∈ S.q do in parallel
        I[j] ← q;
/* initialize T to sequence from 0 to Q.size                                 */
for t ∈ Q.size do in parallel
    T[t] ← t;
/* stable sort T using y-axis (intensity) values of Q (Q.y) as keys          */
StableSortByKey(Q.y, T)
/* gather x-axis (m/z) (Q.x) using T                                         */
gather(Q.x, T);
/* gather I using T                                                          */
gather(I, T);
/* reset T to sequence from 0 to Q.size                                      */
for t ∈ Q.size do in parallel
    T[t] ← t;
/* stable sort T using I as keys                                             */
StableSortByKey(I, T)
/* gatherQ.x and Q.y using T                                                 */
gather(Q.x, T);
gather(Q.y, T);
/* filter only top-K (last K) intense (Q.y) data points                      */
/* ∀q ∈ Q                                                                    */
Q' ← filter(Q.x, Q.y, k);
return Q';
```

3.4 Supplementary Algorithm 4

Algorithm 4: Step 3: GPU Database Peptide Search

Data: experimental spectra batch (Q), CFIR-Index instance (D), tolerances ($\delta F, \delta M$)

Result: top peptide matches (h), null distribution of scores (N)

```
/* compute filtered database peptides lists ∀q ∈ Q */
p_min, p_max ← filter(Q, D, δM, δF);
/* shared memory arrays for reduction */
__shared__ keys[], vals[];
/* search all ions in all spectra */
for q ∈ Q do in parallel
    /* get filtered range for the current q */
    m_min, m_max ← p_min[q], p_max[q];
    for i ∈ q do
        /* compute database fragment-ions f ∈ i ± δF */
        F_lower, F_upper ← computeLimits(m_min, m_max);
        for j ∈ {F_lower, F_upper} do in parallel
            /* compute the matched ion's peptide id */
            keys[tid] ← j/D.len;
            /* write ion-series, intensities etc to shared mem */
            val[tid] ← (j mod D.len)/(D.len/2), i.intn;
            /* reduction loop */
            for k ∈ ⌈(log(activethds))⌉ do
                newKey ← keys[tid + 2^k]
                if newKey = myKey then
                    val[tid] ← val[tid] + val[tid + 2^k];
            /* update global score if group leader */
            if keys[tid] ≠ keys[tid + 1] then
                h[key[tid]] ← h[key[tid]] + val[tid];
    /* compute the null distribution and get max from h */
    for l ∈ h do in parallel
        N[q], h_max[q] ← atomicAdd(N[q], i), reduce(currMax(l));
return h, N;
```

3.5 Supplementary Algorithm 5

Algorithm 5: Step 4: GPU Result Postprocessing

Data: batch of histograms (N) and topscores (h)
Result: batch of expected values (E)

/* shared memory array for probability function (p_x) */
$\_\_shared\_\_p_x[]$
for $n \in N \wedge h \in H$ **do in parallel**
    /* clip the function from first non-zero to top score - 1 */
    $p_x \leftarrow n[largmax(1) : rargmax(1)]$
    /* Use either log-Weibull or Tail Fit */
    if *log-Weibull Fit* **then**
        /* smooth p_x using savitzky-golay filter */
        $p_x \leftarrow savgol(p_x)$;
        for $err > 1^{-3} \wedge n < iter$ **do in parallel**
            /* mean sq error */
            $err \leftarrow \sqrt{(lgW(\mu, \beta) - p_x)^2}$;
            /* update μ and β */
            $\mu, \beta \leftarrow \mu, \beta + fit(\delta\mu, \delta\beta, err)$;
        /* compute e-value */
        $E[n] \leftarrow sum(n) * lgW(\mu, \beta)[h]$;
    else
        /* tail fit */
        /* compute $\log_{10}(cdf)$ */
        $p_x \leftarrow prefixSum(p_x)$;
        $p_x \leftarrow \log_{10}((1 - p_x)/Sum(n))$;
        /* clip the tail of the cdf */
        $p_x \leftarrow p_x[largmax(0.22 * p_x) : rargmax(0.87 * p_x)]$;
        /* linear regression to fit $y = mx + b$ */
        $m, b \leftarrow LinearFit(p_x)$;
        /* compute e-value */
        $E[n] \leftarrow sum(n) * pow(10, m * h + b)$;
return E;

3.6 Supplementary Algorithm 6

Algorithm 6: CUDA Warp-Shuffle based Reductions

Data: thread-local variable (v_l), commutative and associative reduction operation ($\otimes$), conditions $cond1$,$cond2$

Result: reduced global variable v_g

```
/* currently reduced values in v_l                                    */
/* compute the thread mask                                            */
mask ← __ballot_sync(activemask(), cond1);
/* reduce intra-warp                                                  */
for off ∈ warpSize/2, ···, 1 do
    tmp ← __shfl_down_sync(mask, v_l, off);
    if cond2 then
        v_l ← v_l ⊗ tmp;
__shared__ vals[32];
/* The laneId = 0 threads now have the reduced value of their warps   */
if laneId = 0 then
    vals[warpId] ← v_l;
/* read from vals and reduce the first warp only                      */
if warpId = 0 then
    v_l ← vals[laneId];
    /* update thread mask                                             */
    mask ← __ballot_sync(activemask(), warpId = 0);
    for off ∈ warpSize/2, ···, 1 do
        tmp ← __shfl_down_sync(mask, v_l, off);
        if cond2 then
            v_l ← v_l ⊗ tmp;
/* tid = 0 will have the reduced value, broadcast                     */
if tid = 0 then
    vals[0] ← v_l;
/* read and return the reduced value in v_g                           */
return v_g ← vals[0];
```

References

[1] Jimmy K Eng, Ashley L McCormack, and John R Yates. An approach to correlate tandem mass spectral data of peptides with amino acid sequences in a protein database. *Journal of the American Society for Mass Spectrometry*, 5(11):976–989, 1994.

[2] Jimmy K Eng, Bernd Fischer, Jonas Grossmann, and Michael J MacCoss.

A fast sequest cross correlation algorithm. *Journal of proteome research*, 7(10):4598–4602, 2008.

[3] Jimmy K Eng, Tahmina A Jahan, and Michael R Hoopmann. Comet: an open-source ms/ms sequence database search tool. *Proteomics*, 13(1):22–24, 2013.

[4] Sean McIlwain, Kaipo Tamura, Attila Kertesz-Farkas, Charles E Grant, Benjamin Diament, Barbara Frewen, J Jeffry Howbert, Michael R Hoopmann, Lukas Kall, Jimmy K Eng, et al. Crux: rapid open source protein tandem mass spectrometry analysis. *Journal of proteome research*, 13(10):4488–4491, 2014.

[5] Robertson Craig and Ronald C Beavis. Tandem: matching proteins with tandem mass spectra. *Bioinformatics*, 20(9):1466–1467, 2004.

[6] Hao Chi, Kun He, Bing Yang, Zhen Chen, Rui-Xiang Sun, Sheng-Bo Fan, Kun Zhang, Chao Liu, Zuo-Fei Yuan, Quan-Hui Wang, et al. pfind–alioth: A novel unrestricted database search algorithm to improve the interpretation of high-resolution ms/ms data. *Journal of proteomics*, 125:89–97, 2015.

[7] Marshall Bern, Yuhan Cai, and David Goldberg. Lookup peaks: a hybrid of de novo sequencing and database search for protein identification by tandem mass spectrometry. *Analytical chemistry*, 79(4):1393–1400, 2007.

[8] Krste Asanovic, Ras Bodik, James Demmel, Tony Keaveny, Kurt Keutzer, John D Kubiatowicz, Edward A Lee, Nelson Morgan, George Necula, David A Patterson, et al. The parallel computing laboratory at uc berkeley: A research agenda based on the berkeley view. *EECS Department, University of California, Berkeley, Tech. Rep*, 2008.

[9] You Li and Xiaowen Chu. Speeding up scoring module of mass spectrometry based protein identification by gpu. In *2012 IEEE 14th International Conference on High Performance Computing and Communication & 2012 IEEE 9th International Conference on Embedded Software and Systems*, pages 1315–1320. IEEE, 2012.

[10] Lydia Ashleigh Baumgardner, Avinash Kumar Shanmugam, Henry Lam, Jimmy K Eng, and Daniel B Martin. Fast parallel tandem mass spectral library searching using gpu hardware acceleration. *Journal of proteome research*, 10(6):2882–2888, 2011.

[11] Jeffrey A Milloy, Brendan K Faherty, and Scott A Gerber. Tempest: Gpu-cpu computing for high-throughput database spectral matching. *Journal of proteome research*, 11(7):3581–3591, 2012.

[12] You Li, Hao Chi, Leihao Xia, and Xiaowen Chu. Accelerating the scoring module of mass spectrometry-based peptide identification using gpus. *BMC bioinformatics*, 15(1):1–11, 2014.

[13] You Li, Leihao Xia, Hao Chi, and Xiaowen Chu. Accelerating mass spectrometry-based protein identification using gpus. *BMC Bioinformatics*, 2014.

[14] Hyunwoo Kim, Sunggeun Han, Jung-Ho Um, and Kyongseok Park. Accelerating a cross-correlation score function to search modifications using a single gpu. *BMC bioinformatics*, 19(1):1–5, 2018.

[15] Chuang Li, Kenli Li, Keqin Li, and Feng Lin. Mctandem: an efficient tool for large-scale peptide identification on many integrated core (mic) architecture. *BMC bioinformatics*, 20(1):397, 2019.

[16] Hao Chi, Chao Liu, Hao Yang, Wen-Feng Zeng, Long Wu, Wen-Jing Zhou, Xiu-Nan Niu, Yue-He Ding, Yao Zhang, Rui-Min Wang, et al. Open-pfind enables precise, comprehensive and rapid peptide identification in shotgun proteomics. *bioRxiv*, page 285395, 2018.

[17] Andy T Kong, Felipe V Leprevost, Dmitry M Avtonomov, Dattatreya Mellacheruvu, and Alexey I Nesvizhskii. Msfragger: ultrafast and comprehensive peptide identification in mass spectrometry–based proteomics. *Nature methods*, 14(5):513, 2017.

[18] Arun Devabhaktuni, Sarah Lin, Lichao Zhang, Kavya Swaminathan, Carlos G Gonzalez, Niclas Olsson, Samuel M Pearlman, Keith Rawson, and Joshua E Elias. Taggraph reveals vast protein modification landscapes from large tandem mass spectrometry datasets. *Nature biotechnology*, page 1, 2019.

[19] Siegfried Gessulat, Tobias Schmidt, Daniel Paul Zolg, Patroklos Samaras, Karsten Schnatbaum, Johannes Zerweck, Tobias Knaute, Julia Rechenberger, Bernard Delanghe, Andreas Huhmer, et al. Prosit: proteome-wide prediction of peptide tandem mass spectra by deep learning. *Nature methods*, 16(6):509–518, 2019.

[20] Muhammad Haseeb and Fahad Saeed. High performance computing framework for tera-scale database search of mass spectrometry data. *Nature Computational Science*, 1(8):550–561, 2021.

[21] Muhammad Haseeb and Fahad Saeed. Efficient shared peak counting in database peptide search using compact data structure for fragment-ion index. In *2019 IEEE International Conference on Bioinformatics and Biomedicine (BIBM)*, pages 275–278. IEEE, 2019.
